# Asymmetric metamaterial sandwich structure with NIM characteristics for THz imaging application

**DOI:** 10.1038/s41598-024-56723-w

**Published:** 2024-03-15

**Authors:** Tayaallen Ramachandran, Mohammad Rashed Iqbal Faruque, K. S. Al-mugren

**Affiliations:** 1 Space Science Centre (ANGKASA), Institute of Climate Change (IPI), Universiti Kebangsaan Malaysia, 43600 Bangi, Selangor Malaysia; 2 Physics Department, Science College, Princess Nourah Bint Abdulrahman University, Riyadh, Saudi Arabia

**Keywords:** Absorbance, Asymmetric, Negative index metamaterial, Reflectance, Sandwich structure, Silicon, THz imaging, Electrical and electronic engineering, Characterization and analytical techniques

## Abstract

This study presented a unique, miniaturised asymmetric interconnected vertical stripe (IVS) design for terahertz (THz) frequency applications. Therefore, this research aimed to achieve a frequency response of 0 to 10 THz using a 5 × 5 µm^2^ Silicon (Si) substrate material. Meanwhile, various parametric examinations were conducted to investigate variations in the performance. For example, the unit cell selection process was carefully examined by using various design structures and modifying the structure by adding split gaps and connecting bars between vertical stripes. Furthermore, the proposed sandwich structure design was used to compute the absorbance and reflectance properties. All the analytical examinations were executed utilising the Computer Simulation Technology (CST) 2019 software. The introduced IVS metamaterial exhibits negative index behaviour and has a single resonance frequency of 5.23 THz with an acceptable magnitude of − 24.38 dB. Additionally, the quadruple-layer IVS structure exhibits optimised transmission coefficient behaviour between 3 and 6 THz and 7 to 9 THz, respectively. However, the magnitude of the transmission coefficient increased with the number of material layers. Besides that, the absorbance study shows that using a quadruple-layer structure obtains unique and promising results. Overall, the proposed asymmetric IVS metamaterial design achieves the required performance by using a compact structure rather than extending the dimensions of the design.

## Introduction

Terahertz frequency investigations have become famous among researchers all over the world because they possess unique characteristics. Terahertz radiation, for example, is non-ionizing and regarded as safe for humans at low power. Besides that, water molecules absorb it very significantly, which reduces the amount of tissue penetration by hundreds or even tens of microns. Therefore, there is a growing understanding that greater awareness is being given to terahertz metamaterials to be applied in various research areas. Generally, the term metamaterial is defined as an artificial material that cannot be obtained in nature. Hence, conventional metamaterials are widely applied for application fields around the world, including sensors, radar cross-section reduction applications, filters, microwave frequency applications, antennas, and specific absorption rate reduction (SAR) applications^1–13^.

In 2022, Xu et al.^14^ presented tunable active MEMS-based metamaterials for advanced and innovative applications. The authors studied and examined the creation of MEMS-based metamaterials utilising various actuators, including electrostatic, stretching actuation mechanisms, electromagnetic and electrothermal. Meanwhile, Gao et al.^15^ demonstrated broadband vibration attenuation using a rainbow metamaterial beam combined with a graded structure of acoustic black holes. In this article, a popular genetic algorithm and mathematical model are used to statistically evaluate wave propagation behaviours in beam optimisation of finite rainbow metamaterial beams, transmittance characteristics, and infinite metamaterial beams. Moreover, in 2022, Lumin et al.^16^ introduced a new metamaterial that coupled thermal shrinkage and authenticity by including a double-square and bi-material within a shaped like a star configuration in 2022. Meanwhile, a theoretical model based on Mohr's theorem was created to predict its thermal and elastic characteristics. During the same period, Xiang et al.^17^ published a universal but practical design technique focused on the element of topological metamaterial, which enables the stimulation of any high-order spatial mode in Si waveguides.

Furthermore, conventional metamaterials are also applied for various terahertz frequency applications. Samy et al.^18^ proposed and investigated very sensitive absorbers-inspired metamaterial design for THz sensing applications. The proposed design focuses on enhancing the confinement of both magnetic and electric fields at the exhibited peak points. Furthermore, Lee et al.^19^ proposed a biosensing device inspired by a nano metamaterial design for enhancing the cross-section absorption, which is connected to the transmitted terahertz near field augmentation via metamaterial structure. A terahertz actively tunable broadband metamaterial polarisation rotator constructed with two metal and two orthogonal graphene grating devices was proposed and analysed by Zhang et al.^20^ in 2018. This study provides a novel paradigm for researching interactively adjustable THz polarised devices based on graphene materials, which have potential applications in wireless communication and terahertz imaging. Furthermore, Kindness et al.^21^ used graphene for continuous resonance frequency tuning to demonstrate how to actively manage electromagnetically induced transparency in a terahertz metamaterial array.

In addition, the effects of the metamaterial-based rectangular microstrip patch antenna for terahertz applications were studied by Devapriya et al.^22^. The 180 × 212 × 10 μm^3^ sized antenna was proposed by the authors in this work. It is built on a quartz substrate and fed using microstrip line feed technology. Meanwhile, the metamaterial was constructed by adopting the circular split ring resonator design structure. On the other hand, Roh et al.^23^ proposed a much better terahertz imaging method by intuitively comparing complementary metamaterials using Babinet's approach. There is a significant and clear correlation between the polarisation angle and the complementary metamaterials' terahertz reflectance spectrum. Furthermore, Tang et al.^24^ proposed a switchable, functional, and tunable metamaterial device based on hybrid graphene-vanadium dioxide. The adoption of metal–insulator transition features in graphene-vanadium dioxide leads to the proposed metamaterials switching between tunable circular dichroism and dual-band perfect absorption in the terahertz frequency range. A planar hybrid metamaterial device with constant insertion loss across a finite frequency range that can be electrically controlled was presented by Padilla et al.^25^ in 2009. This device can linearly change the phase of terahertz radiation. The device may also serve as a broadband terahertz modulator due to the causal relationship between phase shifting and amplitude modulation.

Moreover, a double-band terahertz frequency metamaterial absorber sensor was introduced by Pang et al.^26^ in 2021. The sensing range of the developed terahertz metamaterial absorber sensor was determined by analysing a series of materials with varied refractive indices. In 2015, Wang et al.^27^ presented a new type of dual-band terahertz metamaterial absorber formed by a patterned metallic strip and a dielectric layer on top of a metallic ground plane. Besides that, Zhao et al.^28^ proposed an optically modulated ultra-broadband with all Si metamaterial for a terahertz-perfect absorber. Terahertz perfect absorbers are a critical photonic component in the detection, modulation, and manipulation of terahertz radiation. In this work, the authors demonstrate tunable ultra-broadband terahertz wave absorption using single-layer H-shaped all Si arrays. In 2020, Zhang et al.^29^ focused on three main objectives, likely proposing an ultra-wideband terahertz metamaterial perfect absorber, adding phase-change material Vanadium Dioxide to improve the structure, and finally, tunable terahertz metamaterial absorbers based on Vanadium Dioxide. On the other hand, in 2023, Sheta et al.^30^ analysed metamaterial-based THz polarisation-insensitive wherein the top metasurface was comprised of MgF_2_-graphene periodic nanopillars formed over an InSb nanolayer. Meanwhile, the same first author^31^ proposed a polarisation-insensitive ultra-wideband metamaterial absorber comprising a zirconium nitride (ZrN) based metasurface. The performance of the suggested structures was studied under a variety of parametric settings, including incidence excitations with transverse electric (TE-) and transverse magnetic (TM-) fields.

Despite the unique properties of metamaterial design in various research fields, an exceptional novel design structure is required for this modern era, which helps to enhance its performance. First of all, the terahertz frequency was selected in this work because the visible and near-infrared areas of many materials are opaque, which severely restricts the scope of additional study possible. Remarkably, one of the most appealing aspects of THz radiation is that these materials continue to be visible in the THz range. The materials will exhibit a stark contrast because of the variation in water content since THz is phase-sensitive to moisture. As an illustration, THz waves may pass through non-polar molecules like paper and plastic to identify packaging content non-destructively without having to open the package. Moreover, the miniaturisation concept is still a major limitation among researchers. This is because reducing metamaterial design typically causes difficulty in obtaining the desired resonance frequency. Therefore, in this research work, several parametric studies were analysed by utilising the trial and error method by adopting a compact metamaterial design. Furthermore, only limited research was performed by adopting a conventional metamaterial design structure for enhancing THz imaging. Typically, the intriguing potential of metamaterials for THz radiation derives from a resonant electromagnetic response that may be altered for specific purposes. Hence, the novel sandwich-shaped asymmetric metamaterial was proposed in this work by satisfying the major constraint for the THz imaging application.

## Unit cell design and numerical simulation methods

The popular Computer Simulation Technology (CST) Studio Suite 2019.00^32^ software was used for all simulation analyses in this study. Before building the actual prototype, the CST software typically generates timely and reliable results for the virtual prototype. This can be tested physically and has shorter development cycles. Additionally, a CPU with an Intel (R) Core (TM) i7-10700 @ 2.90 GHz and 16 GB of RAM was used to run the simulation. Depending on the size of the structure, the simulation took a certain amount of time to complete, with smaller structures needing less time than bigger metamaterials. Overall, the simulations ranged in time from 33 s to 1 h and 14 min. These analytical investigations are organised into three sections: scattering parameters analyses, computation of effective medium parameters, and finally analysis of absorbance and reflectance computations using sandwich structures. CST software was used to compute absorbance, reflectance, and scattering, whereas MathWorks MATLAB R2021a (9.10.0.1602886)^33^ software was used to derive effective medium characteristics. Furthermore, for the scattering parameter evaluation, the simulation in CST used the hexahedral mesh and time-domain solver. For example, the suggested IVS metamaterial design was placed between two waveguide ports to calculate the values of the reflection (S11) and transmission coefficient (S21). These ports were arranged along the z-axis to represent a transverse electromagnetic mode. Additionally, the y-axis was set up as a perfect magnetic conductor, whereas the x-axis was set up as a perfect electric conductor. Meanwhile, the S21 result from this software was verified by adopting Ansys High-Frequency Structure Simulator (HFSS) 15.0^34^.

This study focused mostly on frequency bands that ranged from 0 to 10 THz. As a result, this frequency range has been designated early in the development of metamaterial design. The introduced design was modified through a trial and error method to provide outcomes that met the desired frequency range. At the end of the first simulation phase, the scattering parameters for the proposed design were derived. Afterwards, the S11 and S21 data were used to construct the effective medium parameters. As a result, the Robust method was utilised to calculate various parameters such as permittivity (ε), permeability (μ), and refractive index (n) values using the MATLAB software^35–37^. This software is a powerful and reliable numerical simulation application with a high-level scripting language for scientific and mathematical calculations. The retrieval equations of z, n, ε, and μ are described below by Eqs. (1)–(4).

1 $$z=\pm \surd \frac{{(1+{S}_{11})}^{2}-{S}_{21}^{2}}{{(1-{S}_{11})}^{2}-{S}_{21}^{2}}$$

$$n=\frac{1}{{k}_{0}d}\{[[In\left({e}^{in{k}_{0}d}\right)]"+2m\pi -i[In\left({e}^{in{k}_{0}d}\right)]\mathrm{^{\prime}}\},$$

2 $${e}^{in{k}_{0}d}=\frac{{S}_{21}}{1-{S}_{11}\frac{(z-1)}{(z+1)}}$$

3 $$\varepsilon =\frac{n}{z}$$

4 $$\mu =nz$$

The symbols ‘ and “ in Eq. (2) indicate the real part and imaginary part operators, respectively. The simulation concludes with the computation of absorbance and reflectance values for the proposed designs. Calculating absorbance and reflectance values for the suggested designs is the last step in this analytical simulation process. A hexahedral mesh and a frequency-domain solver were used in CST software to conduct all of these simulations for the sandwich structures. Reflectance is defined as the ratio of reflected radiation to incoming radiation power. Meanwhile, absorption is defined as the ratio of absorbed radiation to incoming radiation power.

**Figure 1:**
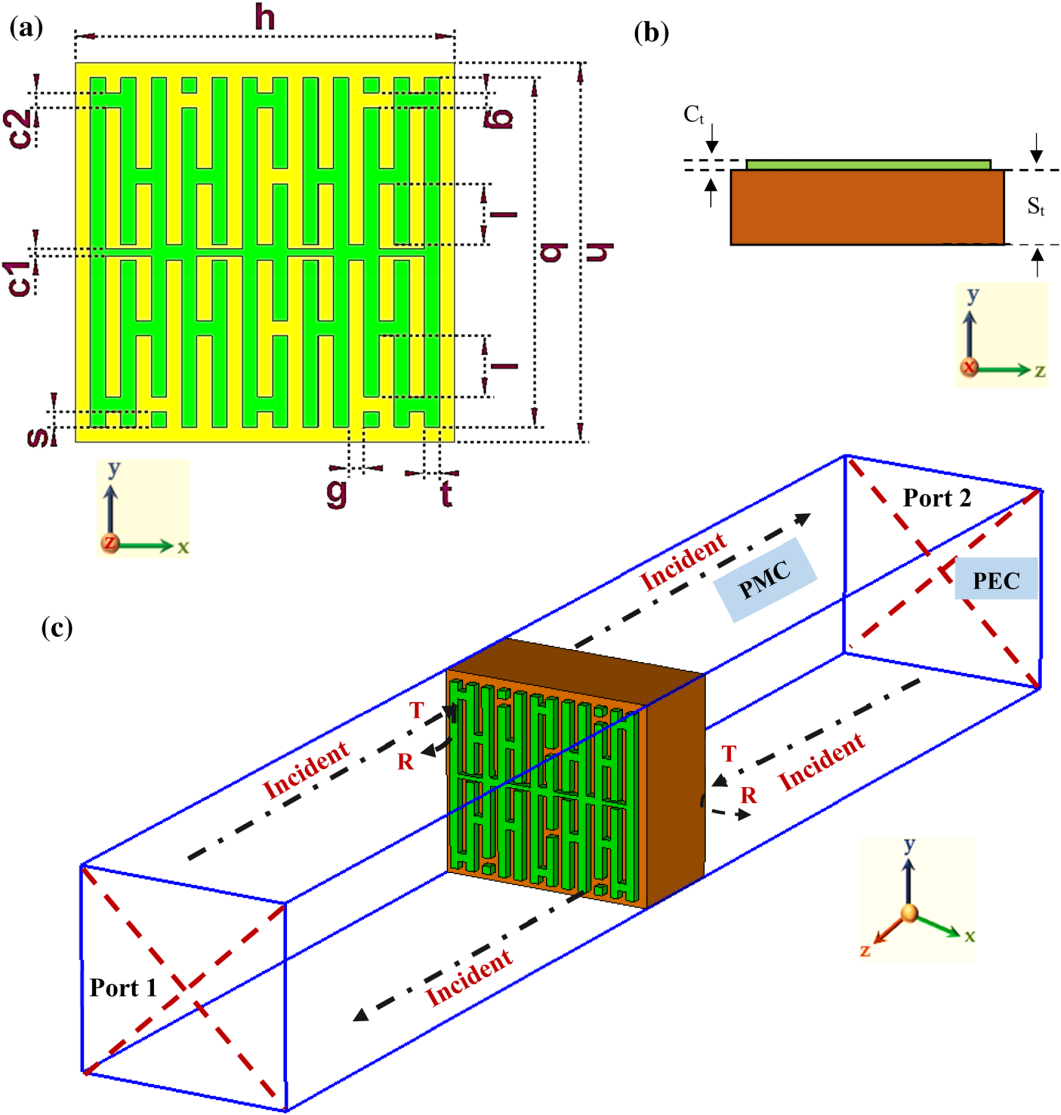
Proposed metamaterial from (**a**) Top view (**b**) Side view; and (**c**) Set up of boundary condition of the proposed IVS metamaterial design.

In this research work, a novel compact asymmetric interconnected vertical stripe (IVS) metamaterial design was proposed. Additionally, the suggested IVS design measures 5 × 5 µm^2^ and uses Si material as the substrate layer with copper serving as the material for the metamaterial frame. Si substrate material has a dielectric constant (ɛ) of 11.9 and a tangent loss (δ) of 0.00025 S/m. In contrast to other materials, Si material doesn't make an ideal conductor or insulator. However, the material is becoming more accessible, making it increasingly common. In other words, Si acts as a substrate for electronics in the modern age. It additionally serves as a common component found in computer chips and microchips. Si material is also more affordable compared to other metals, such as germanium, which might make it harder to form a chip. On the other hand, the relatively low resistivity Si material is easily treated with traditional lithography methods but is potentially considered very lossy in the terahertz band. Twelve vertical stripes with comparable widths (t = 0.2 µm) were built on the substrate material, separated by a g = 0.2 µm, taking into account all of the major THz requirements. In addition, as Fig. 1a illustrates, a split ring gap with a dimension of g was created at various locations. The split gap is a significant limitation when identifying the relevant characteristics. It has something to do with the fact that split gap modifications influence inductance and capacitance values. Overall, variations in the split gaps alter the resonance frequencies, which have a significant effect on the suggested IVS metamaterial. Besides that, a 0.2 µm connecting bar was inserted into the design to link all the stripes. Meanwhile, the connecting bar with a thickness of 0.1 µm was added horizontally at the middle of the design structure to connect the first and last vertical stripe. Overall, the final top view and side view of the proposed design are demonstrated in Figs. 1a,b. Meanwhile, Fig. 1c shows the boundary condition of the introduced IVS metamaterial structure. Table 1 contains a tabulation of all the information about the dimensions of the suggested metamaterial unit cell design.

**Table 1:** Dimensions of the suggested IVS design framework.

Specification	Dimension (µm)
Length & width, *h*	5.0
Copper metamaterial size, (*b* × *b*)	4.6
Gap between structures, *g*	0.2
Width of vertical stripe, *t*	0.2
Small connecting bar, *c*_*1*_	0.1
Big connecting bar, *c*_*2*_	0.2
Vertical stripe bar length, *l*	0.8
Length of square structure, *s*	0.2
Copper thickness, *C*_*t*_	0.2
Si material thickness, *S*_*t*_	2.5

## Scattering and effective medium parameters of IVS design

**Figure 2:**
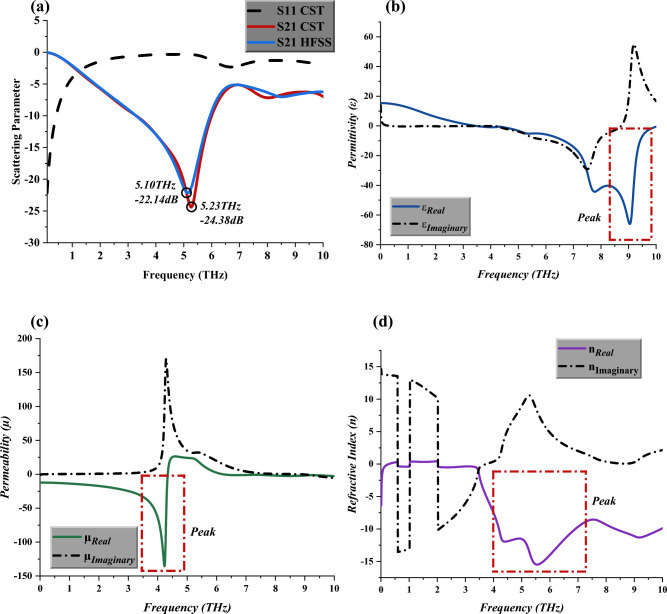
Scattering and effective medium parameters of unit cell metamaterial (**a**) S11 and S21, (**b**) Permittivity, (**c**) Permeability, (**d**) Refractive Index.

The physical characteristics of the suggested IVS metamaterial structure, including scattering and effective medium parameters, are shown in Figs. 2 (a) through (d). Overall, one resonance frequency with magnitudes larger than -15 dB in the region of 0 to 10 THz was found by the simulation using the CST programme. The S21 peak point occurred at 5.23 THz with a magnitude value of -24.38 dB, as shown in Fig. 2 (a). Meanwhile, the S21 result from CST software was verified by using the result from HFSS 15.0 software. The comparison revealed relatively minor differences that were found between both analytical simulation software by approximately 3%. On the other hand, for the S11 behaviour, the proposed design does not manifest any peak response throughout the frequency range. Besides that, the negative permittivity behaviours also occurred in the range of 6 to 9.5 THz, with a peak point that reached almost a -70 magnitude value, as demonstrated in Fig. 2b. Meanwhile, the proposed design possesses negative permeability values below 5 THz, but the resonance peak starts to form in the range of 3 to 4.5 THz. Furthermore, the peak point falls at 4.23 THz with a magnitude value of − 135.64, as demonstrated in Fig. 2c. Finally, Fig. 2d demonstrates the refractive index behaviour where the negative responses focused on the 3 to 10 THz frequency range. Therefore, the proposed asymmetric IVS design can be categorised as a negative index metamaterial (NIM). Achieving a NIM was significant since this feature is uncommon in natural materials, and such metamaterials helped enhance optical transformations and photonics.

## Parametric studies

In this section, three main analysis investigations were performed, such as the unit cell selection process, sandwich structure analysis, and absorbance and reflectance analysis of the proposed design. All the analytical simulations of these analyses were conducted using similar CST software. Initially, five styles of metamaterial designs were suggested for the unit cell selection process by adopting the trial and error method. Afterwards, the optimised design from the five structures was selected to further explore the S21 performances by altering the metamaterial design. Meanwhile, the introduced IVS metamaterial was arranged in a sandwich structure such as double, triple and quadruple layers. Finally, the absorbance and reflectance properties of the sandwich structure are also investigated in this work. Overall, these parametric studies exhibit unique performance changes when compared to proposed unit cell designs.

### Selection process of unit cell design

**Figure 3:**
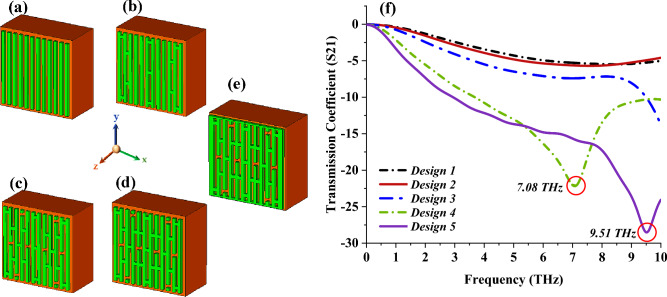
Unit cell designs (**a**) Design 1, (**b**) Design 2, (**c**) Design 3, (**d**) Design 4, (**e**) Design 5, (**f**) S21.

During the primary state of design construction, the Si substrate material and copper metamaterial structure were adopted. Meanwhile, 12 vertical stripe structures were placed on the substrate material, as shown in Fig. 3a. Moreover, split gaps and connecting bars with specific dimensions were placed randomly by adopting the trial and error method, and four of the best designs were selected as shown in Figs. 3b–e. The S21 behaviours of the selected structures were graphed in Fig. 3f, and the observation revealed key findings where Designs 4 and 5 exhibit single resonance at 7.08 and 9.51 THz, respectively. Meanwhile, the first three design structures did not manifest any resonance peak, and only slight curves occurred around zero. Overall, Design 4 was selected for the next parametric analysis since it manifests a peak point at lower bands when compared to Design 5.

**Figure 4:**
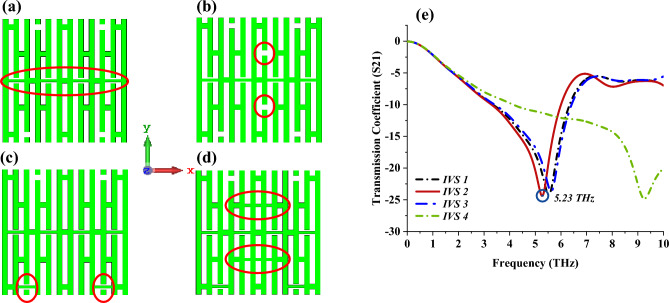
Outcomes of further analysis of adopted Design 4 by adding split gaps and connecting stripe bars (**a**) IVS 1, (**b**) IVS 2, (**c**) IVS 3, (**d**) IVS 4, (**e**) S21.

Besides that, the selected design from the previous parametric study was adopted to further investigate the S21 behaviours when additional connecting bars and split gaps were added to Design 4, as demonstrated in Fig. 4a–d. Whereas, the changes made to the design were circled in red colour. The results highlight that another promising response in lower bands was exhibited at 5.23 THz by IVS 2, as shown in Fig. 4e. On the other hand, IVS 1 and 2 designs exhibit almost equivalent responses and have slight discrepancies in S21 values when compared to IVS 2. However, the additional 0.1 µm connecting bar as shown in Fig. 4d causes the S21 values to shift to a higher band. Overall, the IVS 2 design was selected due to its response in the lower band with acceptable magnitude values near -25 dB.

### Sandwich structure design

**Figure 5:**
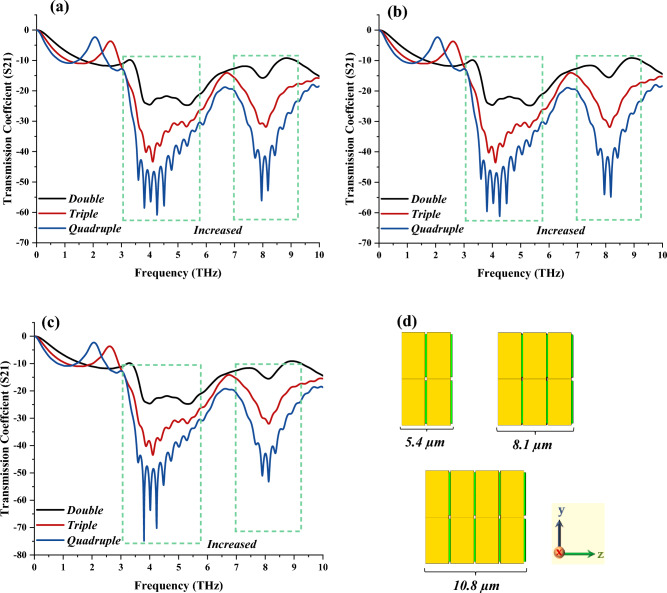
Sandwich IVS 2 metamaterial structure of (**a**) Unit cell, (**b**) 2 × 1 array, (**c**) 2 × 2 array, (**d**) Sandwich structure thickness.

Figure 5a–c demonstrate the S21 behaviours of three types of cell designs. Two types of sandwich structures were constructed in this parametric study as demonstrated in Fig. 5d. First of all, the adopted IVS 2 was designed in a sandwich structure for unit cell, 2 × 1 array cell, and 2 × 2 array cell designs. In each cell design, three layers were utilised, such as the double, triple, and quadruple layers, by using a similar design structure in every layer. The overall S21 result observation revealed almost similar response patterns with slight discrepancies between them. For instance, the number of resonance frequencies was increased at the same frequency range, likely at 3 to 6 THz and 7 to 9.5 THz when the number of layers increased. However, the 2 × 2 array cell design for the quintuple layer structure manifests the highest peak point at 3.8 THz with a magnitude value of − 74.85 dB. Meanwhile, unit cell and 2 × 1 array cell designs manifest multiple resonance frequencies with magnitude values almost near − 60 dB. In a nutshell, the compact unit cell design can be modified by adding layer structures instead of the overall dimension, which leads to a great change in the behavioural performances.

**Figure 6:**
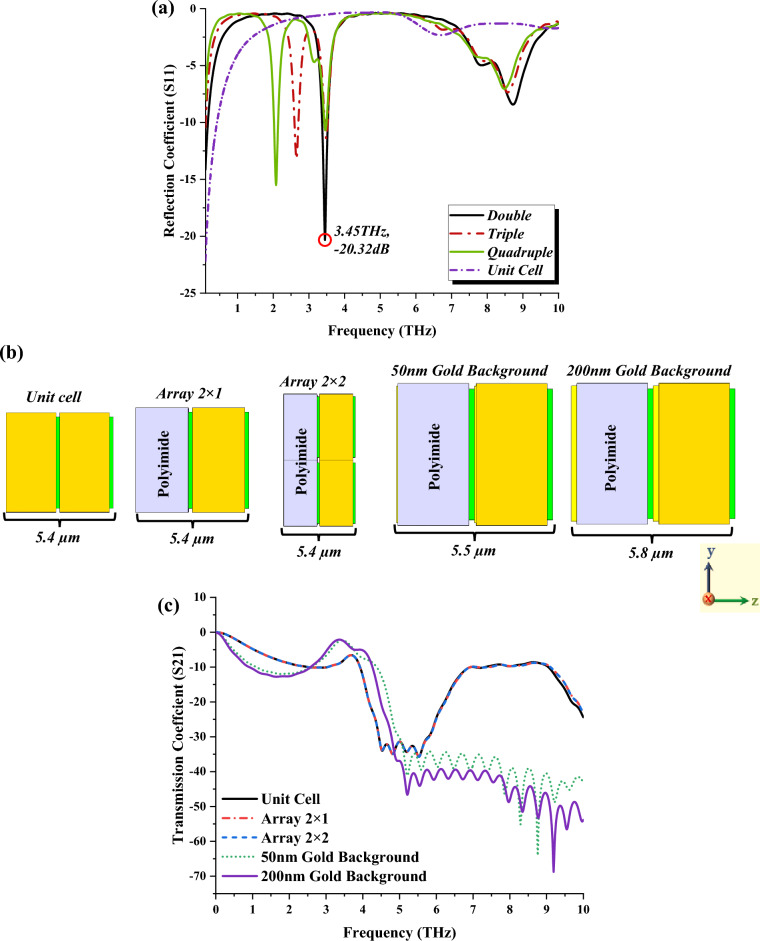
(**a**) S11 of sandwich structure analysis, (**b**) Dimension of various unique sandwich structures, (**c**) S21 of these unique metamaterial designs.

Meanwhile, the S11 behaviours of the introduced IVS metamaterial are also explored, as illustrated in Fig. 6a. The comparison revealed that the unit cell design failed to manifest resonance frequencies within the range. However, all the sandwich structures produce at least one single peak point with an acceptable magnitude value. For instance, double layer design manifests the optimal resonance frequency at 3.45 THz with a magnitude value of − 20.32 dB. On the other hand, the quadruple-layer design exhibits multiple responses with limited magnitude values at 2.08, 3.46, and 8.47 THz. On this basis, we conclude that the number of layer increments directly influences the S11 behaviour in contrast to the outcomes of unit cell design.

Overall, the sandwich structure analysis of the IVS 2 design manifested almost similar outcomes in distinct frequency ranges. Therefore, for the second phase, two types of substrate material were adopted to further explore the changes in S21 behaviour. For instance, the polyimide substrate material with similar thickness was placed behind the Si substrate material and utilised the same metamaterial design as demonstrated in Fig. 6b. Moreover, the array structure was also adopted to compare the performances with a maximum of 2 × 2 array cell designs. Besides that, the gold material layers with thicknesses of 50 nm and 200 nm were placed behind each substrate material. This causes the design dimension to increase by 0.1 and 0.4 µm when compared to the double-layer unit cell design. The first three designs in Fig. 6b exhibit identical short multiple responses with magnitude values near -40 dB. However, adding a gold layer on the backside of the design structure causes the S21 values to change, as shown in Fig. 6c. Therefore, it proves that the combination of a sandwich structure and a gold background can lead to unique performances.

### Absorbance and reflectance analysis

**Figure 7:**
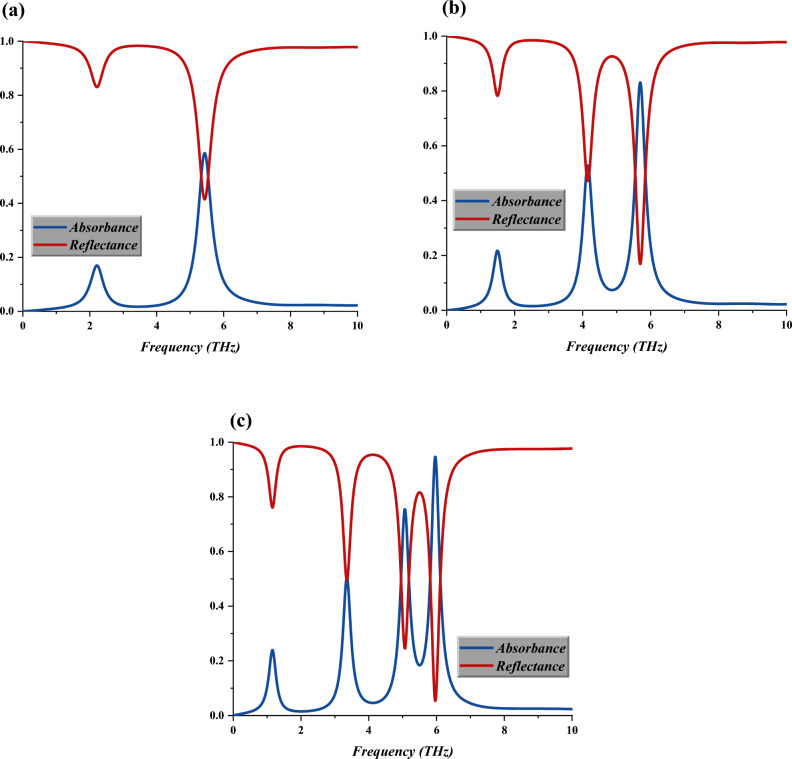
Absorbance and reflectance analysis of (**a**) Double layer, (**b**) Triple layer, (**c**) Quadruple-layer.

Figure 7a–c illustrate the absorbance and reflectance values of the selected IVS 2 for double, triple, and quadruple layers. Superior results are seen from the observation where the absorbance values increase as the number of layers modified from double to quadruple. Moreover, the double-layer design only manifests a single absorbance value at 5.43 THz with an amplitude value of 0.59. Besides that, the number of absorbances increases to two or more as the IVS 2 design is constructed in double and quadruple layers. For instance, the quadruple-layer design exhibits four absorbance peak points at 1.17, 3.35, 5.07, and 5.96 THz with amplitude values of 0.24, 0.51, 0.75, and 0.95, respectively.

## Polarisation analysis

**Figure 8:**
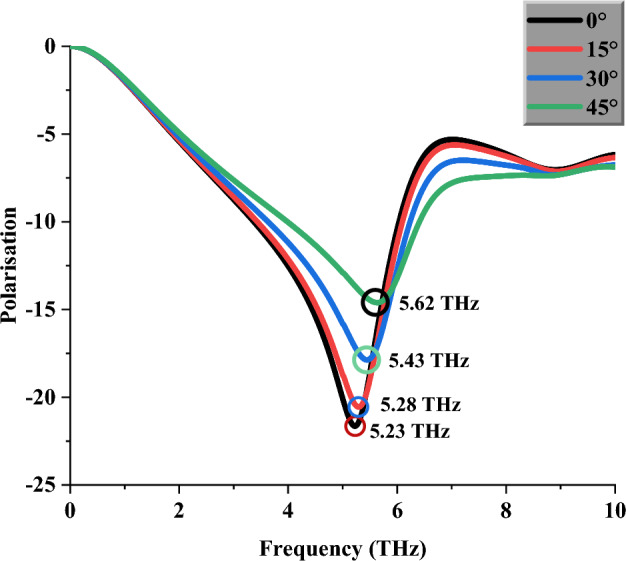
Polarisation-dependent characteristic of S21 value for proposed IVS design.

Figure 8 illustrates the S21 of the introduced unit cell asymmetric structure under various polarisations of 0°, 15°, 30°, and 45°. Polarisation is a feature of transverse waves that determines the geometric orientation of the oscillations. These numerical simulations were also performed with similar CST software. This analysis revealed slight discrepancies occurred between each polarisation value, with a maximum of 7.46%. Therefore, the proposed IVS metamaterial is declared to have a polarisation-dependent characteristic. Besides that, Table 2 illustrates the comparison of several recently published metamaterial designs for terahertz frequency^38–42^ and the proposed asymmetric IVS metamaterial. The overall observation revealed that most of the literature works adopted multiple materials as substrates and for the metamaterial design structures, except^39^. For example, research works from ^38,40,41^ constructed substrate materials by adopting two types of materials, such as insulators, gold, polymide, TOPAS, and Si. Meanwhile, the observation also shows that the proposed design was constructed by adopting compact dimensions when compared to the literature review. However, the major drawback was faced when designing a smaller metamaterial since it is difficult to gain the desired responses. Hence, by adopting extensive analysis, the optimised metamaterial structure was successfully selected for the THz imaging application.

**Table 2:** Comparison table of previously published works with the proposed IVS metamaterial.

Reference	Frequency (THz)	Size (µm × µm)	Substrate	Metamaterial design	Application
^38^	1 to 10	20 × 20	Insulator and Gold	Vanadium oxide and Graphene	Absorber
^39^	0.4 to 2	100 × 100	Si	Gold	Sensing
^40^	3 to 10	40 × 40	Polymide and Gold	Vanadium oxide and Gold	Absorber
^41^	1 to 7	15 × 15	TOPAS and Gold	Graphene	Absorber
^42^	1 to 10	9 × 9	Silica, Gold and Si	Graphene	Absorber
IVS metamaterial	0 to 10	5 × 5	Si	Copper	THz imaging

## Conclusion

In conclusion, the research argues that by using a compact asymmetric metamaterial sandwich structure, the necessary application features may be achieved for THz imaging. Given the widespread use of miniaturisation principles in various research areas in our modern era, it is typically viewed as a positive and novel element for this work. As a result, the proposed compact IVS metamaterial design accomplishes the goal and demonstrates a distinct NIM behaviour. Besides that, several perturbs design variables were performed to determine the ideal metamaterial structure. For example, the outcomes of these investigations show innovative performance in which the S21 response at a higher frequency changed to a lower frequency by using additional split gaps and connecting bars on the initial design structure. Furthermore, the absorbance analysis conducted in this study revealed novel results by simply changing the unit cell design to sandwich structures. In a nutshell, the suggested asymmetric IVS metamaterial contributes to the acquisition of different characteristics and is appropriate for the THZ imaging application to improve performance.

## Data Availability

The datasets used and/or analysed during the current study are available from the corresponding author on reasonable request.
